# Chloroquine, hydroxychloroquine and COVID-19

**DOI:** 10.1080/24734306.2020.1757967

**Published:** 2020-04-30

**Authors:** T. B. Erickson, P. R. Chai, E. W. Boyer

**Affiliations:** a Division of Medical Toxicology, Department of Emergency Medicine, Brigham and Women’s Hospital, Harvard Medical School, Boston, MA, USA;; b Harvard Humanitarian Initiative, Harvard University, Cambridge, MA, USA;; c The Fenway Institute, Boston, MA, USA;; d Koch Institute for Integrated Cancer Research, Massachusetts Institute of Technology, Cambridge, MA, USA;; e Department of Psychosocial Oncology and Palliative Care, Dana Farber Cancer Institute, Boston, MA, USA

**Keywords:** Chloroquine, hydroxychloroquine, coronavirus, diazepam, epinephrine

## Abstract

The media have featured the antimalarials chloroquine (CQ) and hydroxychloroquine (HCQ) to treat coronavirus (COVID-19). Political leaders have touted their use and recommended availability to the public. These anti-inflammatory agents have substantial human toxicity with a narrow therapeutic window. CQ and HCQ poisoning cause myocardial depression and profound hypotension due to vasodilation. Bradycardia and ventricular escape rhythms arise from impaired myocardial automaticity and conductivity due to sodium and potassium channel blockade. With cardiotoxicity, ECGs may show widened QRS, atrioventricular heart block and QT interval prolongation. CQ may also cause seizures, often refractory to standard treatment. Of concern is pediatric poisoning, where 1–2 pills of CQ or HCQ can cause serious and potentially fatal toxicity in a toddler. The treatment of CQ/HCQ poisoning includes high-dose intravenous diazepam postulated to have positive ionotropic and antidysrhythmic properties that may antagonize the cardiotoxic effects of CQ. Infusions of epinephrine titrated to treat unstable hypotension, as well as potassium for severe hypokalemia may be required. Current scientific evidence does not support treatment or prophylactic use of these agents for COVID-19 disease. Regulatory and public health authorities recognize that CQ/HCQ may offer little clinical benefit and only add risk requiring further investigation before wider public distribution.

A drug called chloroquine, and some people would add to it hydroxychloroquine … now this is a common malaria drug. It’s been around for a long time, so we know if things don’t go as planned, it’s not going to kill anybody.

(United States President, Donald Trump, March 19, 2020) [1]

On the contrary, antimalarials have substantial human toxicity. Chloroquine, with its narrow therapeutic window and irreversible side effects, has a global reputation as a “suicide drug” [2,3]. Hydroxychloroquine, a less toxic derivative of chloroquine has similar structural, therapeutic, pharmacokinetic and toxicological properties [3,4] (Figure 1). Chloroquine has been used since 1940 for the treatment and prevention of malaria. It has also been used to treat amebiais. Both chloroquine and hydroxychloroquine have anti-inflammatory properties and are used to treat autoimmune disorders such as lupus and rheumatoid arthritis.

Chloroquine and hydroxychloroquine poisoning can cause myocardial depression and vasodilatation culminating in profound hypotension. Bradycardia and ventricular escape rhythms arise from impaired myocardial automaticity and conductivity due to sodium and potassium channel blockade. Importantly, the interval between exposure and cardiac arrest in overdose may be in less than 2 h. Due to drug-induced cardiotoxicity, electrocardiograms (ECG) may show a widened QRS complex, atrioventricular heart block, QT interval prolongation as well as U waves from hypokalemia. Chloroquine may also cause seizures, which may be refractory to standard treatment. Of particular concern is pediatric poisoning, where 1–2 pills of chloroquine or hydroxychloroquine can cause serious and potentially fatal toxicity a 10 kg toddler who ingests a pill from exploratory behavior [5,6]. In therapeutic doses, chloroquine and hydroxychloroquine can also cause hemolysis in those with genetic enzyme disorders such as porphyria or glucose-6-phosphate dehydrogenase (G6PD) deficiency.

Poisoning from antimalarials is not just a historical artifact, and the medical messaging on March 19 may have contributed to a spate of recent poisonings. On March 21, 2020 two people in Nigeria were hospitalized following hydroxychloroquine overdose [7]. On March 23, 2020, a man and his spouse presented to a U.S. hospital emergency department in Arizona after ingesting chloroquine phosphate in an apparent attempt to prevent corona virus [8]. The man died soon after ingesting the agent which is commonly used to clean fish aquariums and treat parasitic infections. Community pharmacists have also reported a recent spike in new prescriptions for hydroxychloroquine, specifically many of which were inappropriately written by prescribers for themselves and their families [9].

The treatment of chloroquine and hydroxychloroquine poisoning includes diazepam, a commonly used benzodiazepine which therapeutically acts as a sedative and anticonvulsant, but in higher doses, appears to have dose-related positive ionotropic and antidysrhythmic properties that may antagonize the cardiotoxic effects of chloroquine. The specific mechanism of action of diazepam at these higher doses remains unclear. Consequently, a bolus dose of 1–2mg/kg intravenous diazepam, followed by a continuous infusion at a rate of 1–2mg/kg/hr diazepam, has been recommended [2,10]. Cases with refractory hypotension can be managed by titrating a continuous epinephrine infusion (initiated at 0.25 mcg/kg/min) to maintain blood pressure [2,10]. Potassium supplementation may also be required [2,11]. Repletion, however, needs to be cautious in order to avoid rebound hyperkalemia as the hypokalemia is due to an intracellular shift, not depletion. While most hospitals stock sufficient epinephrine on their cardiac arrest resuscitation carts, few hospitals have adequate supplies of intravenous diazepam [12] that might be needed for unstable patients presenting after acute chloroquine overdose.

Current evidence does not support treatment or prophylactic use of chloroquine or hydroxychloroquine for COVID-19 disease. When asked about the use of hydroxychloroquine to treat COVID-19, the FDA commissioner said, “The FDA’s responsibility to the American people is to ensure that products are safe and effective. We want to do that in the setting of a large, pragmatic clinical trial to actually gather that information.” Unfortunately, clinical trials appear to lack sufficient rigor to provide evidence-based guidance. A recent French study enrolled confirmed COVID-19 patients into in a single arm protocol to receive 600 mg of hydroxychloroquine daily [13]. Viral load in nasopharyngeal swabs of study participants was tested daily in a hospital setting. Depending on clinical condition, azithromycin was added to the treatment protocol. Despite a small sample size and the attrition of a substantial number of participants, the authors claimed the hydroxychloroquine treatment was significantly associated with viral load reduction or disappearance in COVID-19 patients and its effect was potentiated or “reinforced” when azithromycin was added to the treatment regimen. This study has several limitations, including a small sample size (36), limited long-term outcome follow-up, dropout of six patients from the study with exclusion of the sickest patients, non-uniform use of azithromycin, and lack of a randomized or true control group. The QT prolongation caused by these antimalarial drugs may also be exacerbated by the co-administration of azithromycin, particularly in ill patients with preexisting cardiovascular disease or patients with electrolyte imbalances [14].

Leading regulatory and public health authorities recognize that chloroquine and hydroxychloroquine may not be effective in this context and offer little to no benefit. According to the Commissioner of the FDA, “What’s also important is not to provide false hope. We may have the right drug, but it might not be in the appropriate dosage form right now, and it might do more harm than good.” [15] When asked about chloroquine last month, the Lead for Clinical Management of the World Health Organization said “there is no proof that this is an effective treatment at this time. We recommend that therapeutics be tested under ethically approved clinical trials to show efficacy and safety.” [16] We strongly believe that further proclamations from the Executive Branch that do not comport with medical evidence should be avoided. Because if there is no benefit to a therapy, then there can only be risk.

## Figures and Tables

**Figure 1.**
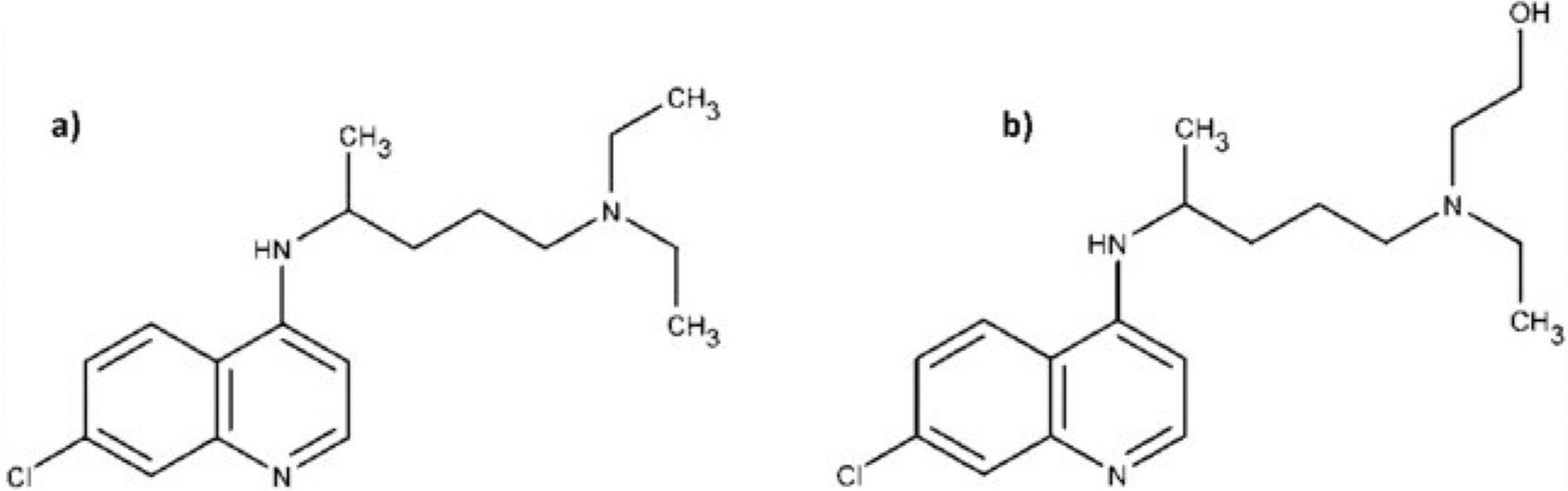
(a) Chloroquine (b) hydroxychloroquine.
